# Public health round-up

**DOI:** 10.2471/BLT.23.010923

**Published:** 2023-09-01

**El Niño weather concerns**

Women fetch water from the Tana River, east of Nairobi, Kenya – one of many countries likely to be impacted by a developing El Niño weather event that is expected to drive an increase in global warming. On 4 August, the World Health Organization’s Department of Alert and Response Coordination reported that the event is highly likely to have wide-ranging health implications for vulnerable populations, notably in terms of food security.

**Figure:**
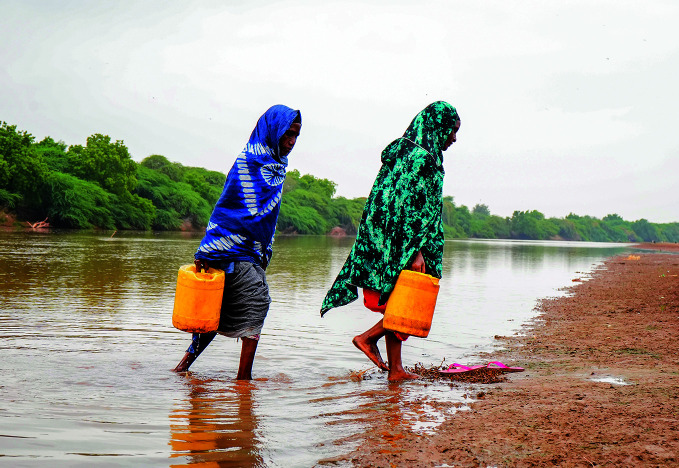
WHO/ Billy Miaron

## The El Niño threat to health

The World Health Organization’s (WHO) Department of Alert and Response Coordination reported on a developing El Niño weather event that is predicted to have wide-ranging implications for public health worldwide.

Published on 4 August, and covering the period July–September 2023, the report draws on research by WHO, the World Meteorological Organization and other partners, and sets out current and potential health challenges faced by vulnerable populations.

The report notes that, in the coming months, the most severe health threats are likely to arise from increased food insecurity, diarrhoea and malnutrition, especially in drought-affected regions.

In many of the countries likely to be most affected, crises are already ongoing, and WHO has emergency response plans and pre-positioned supplies, including supplies for cholera and kits needed to set up stabilization centres to treat severely malnourished children.

In response to the advance warnings around El Niño, WHO will increase emergency stocks where needed and will work with partners to further strengthen local surveillance systems, train medical staff, ensure availability of essential health services including through provision of temporary or mobile clinics. The Organization will also coordinate the response of humanitarian partners.

https://bit.ly/3DSXC7t

## Dengue surge in Bangladesh

The Ministry of Health and Family Welfare of Bangladesh reported a surge in dengue cases since the beginning of the year. As of 7 August, 69 483 laboratory-confirmed dengue cases and 327 dengue-related deaths had been reported, representing a case fatality rate of 0.47%. Some 63% of the cases and 62% of the deaths were reported in the month of July.

Although dengue is endemic in Bangladesh, the surge is unusual in terms of seasonality and the early sharp increase in cases. According to a pre-monsoon survey of the Aedes mosquito populations in Bangladesh, the density of mosquitoes, and the number of potential dengue hotspots are at their highest levels in five years.

https://bit.ly/3QBj5JI

## Africa strengthens disease surveillance

The Africa Centres for Disease Control and Prevention, WHO and the Robert Koch Institute launched the health security partnership to strengthen disease surveillance and epidemic intelligence in Africa.

Announced on 18 July, the initiative aims to strengthen the continent’s health security capacity in the areas of biosecurity, integrated disease surveillance, event-based surveillance, genomic surveillance and epidemic intelligence.

In its first phase, the initiative will be implemented in six countries, Gambia, Mali, Morocco, Namibia, South Africa and Tunisia, and will later be expanded.

https://bit.ly/3YF8hMU

## Ending the war in Sudan

Humanitarian leaders called for an immediate cessation of hostilities in Sudan, where more than four million people have fled the fighting that has been raging for the past four months.

In a communiqué issued on 15 August, the leaders of the humanitarian organizations working in Sudan called on the parties to the conflict to protect civilians and grant safe and unfettered access to them, pointing out that attacking civilians, looting humanitarian supplies, targeting aid workers, civilian assets and infrastructure, including health centres and hospitals, and blocking humanitarian assistance – all of which have been reported in Sudan – are prohibited under international humanitarian law and international human rights law.

The leaders also called on the international community to step up financial support for humanitarian assistance, pointing out that two appeals totalling more than US$ 3 billion were less than 27% funded.

https://bit.ly/45pTVCJ

## New guidance on Ebola and Marburg

WHO issued new guidelines for infection prevention and control of Ebola disease and Marburg disease.

Published on 11 August, the guidance is intended to protect patients, staff and visitors wherever health services are delivered and covers interventions ranging from the implementation of infection prevention and control rings when cases are identified, to the safe handling of individuals dying from the diseases.

https://bit.ly/45sMjyy

## Leveraging traditional medicine

WHO convened the first global summit on traditional medicine. Hosted by the Government of India on 17 and 18 August in in Gandhinagar, Gujarat, India, the summit was attended by the WHO Director-General and Regional Directors; G20 health ministers and high-level invitees from countries across WHO’s six regions; scientists; practitioners of traditional medicine; health workers and members of civil society organizations.

https://bit.ly/454dHn5

## Tackling neurological disorders

WHO released an intersectoral action plan to improve access to care and treatment for people living with epilepsy and other neurological disorders.

Despite the high global burden of disease imposed by neurological conditions, access to both services and support for people impacted is insufficient, especially in low- and middle-income countries.

Developed by WHO in consultation with Member States and other key stakeholders, including people living with neurological disorders, and launched on 20 July, the Intersectoral Global Action Plan 2023 sets out the actions needed as part of a comprehensive, coordinated response across sectors.

https://bit.ly/3DSY85p

## Essential medicines for alcohol use disorders

WHO added acamprosate and naltrexone – two medicines used in the treatment of alcohol use disorders in adults – to the latest edition of the *Model List of Essential Medicines* which was published on 26 July.

Aligned with WHO recommendations on the management of alcohol use disorders, the decision to include the medicines reflects the significant public health burden associated with alcohol use disorders, and will increase the treatment options available to patients and clinicians.

https://bit.ly/442NuUB

**Cover photo**

A Building Foundation for Development staff member helps prepare supplies for delivery to health facilities and displacement camps in Al Jazirah, Sudan, which is currently hosting internally displaced persons fleeing conflict in Khartoum, Sudan.

**Figure:**
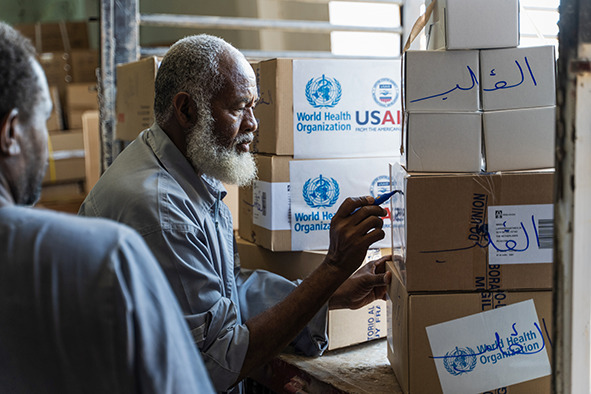
(WHO/Ala Kheir)

## Iraq trachoma achievement

Iraq eliminated trachoma as a public health problem, the fifth country in WHO’s Eastern Mediterranean Region to do so. In a 31 July statement, WHO outlined the main drivers of the country’s success which include establishing a national trachoma programme in 2012 to coordinate the final push against the disease. 

https://bit.ly/3DVgFOI

## One life, one liver

WHO called for the scaling up of testing and treatment for viral hepatitis, warning that, if current infection trends continue, the disease could kill more people than malaria, tuberculosis, and HIV combined by 2040.

Issuing the call on 28 July, World Hepatitis Day, WHO drew attention to the fact that only 1 in 5 of the estimated 58 million people living with hepatitis C infection are diagnosed, while just over 1 in 10 receive treatment. The picture is even bleaker regarding hepatitis B, with only 1 in 10 of the estimated 300 million people living with the virus diagnosed, and only 2% receiving medication.

https://bit.ly/449CmFs

## Abortion care app launched

WHO released a digital decision support tool in the form of an application based on WHO abortion care guidance to support caregivers providing comprehensive abortion care.

Based on the individual characteristics of the patient, the tool generates specific assessments or recommendations which can then inform health-care providers’ decision-making. The tool also provides support for carers by flagging possible patient risks, providing checklists and scheduling individualized post-abortion follow-ups and referrals.

https://bit.ly/3DVFWZ4

**Looking ahead**

20 September 2023. United Nations High-Level Meeting on Pandemic Prevention, Preparedness and Response. UN HQ, New York, United States of America. https://bit.ly/447mO51

21 September 2023. United Nations High-Level Meeting on Universal Health Coverage. UN HQ, New York, United States of America. https://bit.ly/447mO51

22 September 2023. United Nations High-Level Meeting on the fight against tuberculosis. UN HQ, New York, United States of America. https://bit.ly/447mO51

